# Editorial: “Unravelling micro-/nano-plastics toxicity profiling: can we link associated effects to intrinsic characteristics?”

**DOI:** 10.3389/ftox.2025.1605402

**Published:** 2025-04-14

**Authors:** Sebastian Beggel, Valentina Helen Pauna, Vanessa Modesto, Marisa Sárria Pereira de Passos

**Affiliations:** ^1^ Aquatic Systems Biology Unit, TUM School of Life Sciences, Technical University of Munich, Freising, Germany; ^2^ Norwegian Institute for Sustainability Research (NORSUS), Fredrikstad, Norway; ^3^ Lehrstuhl für Biotechnologie, RWTH Aachen University, Aachen, Germany

**Keywords:** microplastics, nanoplastics, exposure assessment, intrinsic toxicity, mechanism of toxicity, in vitro methods, one health, environmental impact and risks

## Introduction

Micro- and nanosized plastic particulates (MNPs) are a diverse group of synthetic substances recognized as an increasing environmental problem (MacLeod et al., 2021; Weis and Alava, 2023). Due to the heterogenic nature, with respect to emission pathways, particle size, polymer type and interactions with other environmental chemicals, this substance group requires novel approaches to better understand the diverse range of impacts on ecosystem health (Beggel et al., 2025; Koelmans et al., 2022). For a sound risk and impact assessment, it is essential to gain more knowledge on their environmental transport, fate and degradation behavior. Data gaps which are particularly key to be tackled include processes that determine MNPs bioavailability and exposure scenarios towards biota as well as the application of meaningful test endpoints that allow the characterization of the variety of potential effects, especially on the sublethal level.

This Research Topic highlights four manuscripts that exemplarily cover the latest scientific developments and findings on the toxicology of micro- and nanoplastics exposures with specific focus on new test-methods and approaches allowing for a sensitive and in-depth environmental fate and effect assessment.

The first manuscript, “Modeling marine microplastic emissions in Life Cycle Assessment: Characterization factors for biodegradable polymers and their application in a textile case study,” by Pellengahr et al. integrates primary experimental data and modelling approaches to determine the fate factors of biodegradable polymer fibers released from textiles. The authors demonstrated that the derived characterization factors are only applicable for physical effects on biota, largely due to data availability, while acknowledging that degradation products of certain polymers can result in toxic effects. Thus, the study also highlights the further need for including plastic additive leaching in effect studies, and the consideration of degradation rates in sediments to fill existing knowledge gaps and improve characterization factors for life cycle impact assessment (LCIA).

The second manuscript, “From the ocean to our kitchen table: Anthropogenic particles in the edible tissue of U.S. West Coast seafood species,” by Traylor et al., highlights the complexity of identifying artificial particle sources in aquatic environments. The study provides proof that commercially valuable finfish can be a potential source of particle exposure to humans via food. It is further highlighted that the focus on model species may under-represent the effects on wild species or environmentally relevant MNP concentrations, which may mis/over-represent effects.

The third article, “Behavioral and molecular effects of micro and nanoplastics in fish: Weathered microfibers induce a similar response to nanosized particles,” by Hutton et al. highlights the variable effects of MNPs in fish, and emphasizes the importance of considering particle morphology and size in toxicity studies. By combining different ecotoxicological endpoints on the molecular, behavioral and individual level, the relative toxicities of different particles could be determined. The study provides ecologically relevant data on how weathering can alter the mechanism of toxicity, which is particularly relevant for future risk assessment.

The fourth article, “The heart of plastic: utilizing the *Drosophila* model to investigate the effects of micro/nanoplastics on heart function,” by Hohman et al., fills an important scientific knowledge gap in the toxicological research of MNPs in terrestrial systems. The study highlights that innovative tools studying physiological responses are well suited to characterize sublethal effects after oral plastic particle exposure. Furthermore, the results suggest that sex-specific differences should be considered as a non-negligible- factor in particle toxicity assessment.

## The need for considering complexity and natural variability in MNPs risk and impact assessment

Most of the published research that has investigated possible ecological effects of MNPs over the last decade has employed procedures involving exposure to substances in controlled scenarios to determine uptake and effects in biota, which do not fully reflect what happens in the environment. Often, concentrations tested are not environmentally relevant, under-emphasizing the effects on biota; not considering possible synergistic or antagonistic effects of MNPs mixtures to concomitant particulate agents, contaminants or biota; and only focusing on one trophic level ignoring possible exchanges along trophic chains and within food-webs and, thus, their long-term associated effects. Currently, there is a growing understanding that the complexity of MNPs in the environment needs to account for the variety of particle characteristics (polymer type, size, morphology, hydrophobicity, density, and a growing list of other relevant attributes) and the degradation (weathering) stage, and cannot be limited to a small number of model organisms. To advance our understanding of MNP toxicity and apply this knowledge to risk and impact assessment, future research must move beyond controlled laboratory settings and embrace ecologically realistic approaches that reflect environmental complexity, long-term and multi-stressor effects. Integrating interdisciplinary collaborations and innovative methodologies will be key to link the intrinsic characteristics of these particles to their ecological consequences. In line with the European Commission’s priorities on plastics, a paradigm shift toward holistic, ecosystem-based research is essential to generate robust data, refine risk assessments, and support the development of science-based regulatory thresholds for MNPs.

## Conclusion

The articles published in this Frontiers in Toxicology Editorial Research Topic illustrate the critical role of effective, mechanistic and evidence-based assessments of the ecotoxicological effects of MNPs. This Research Topic is one example of many emerging research initiatives showing how distinguishing intrinsic toxicity and physical effects associated with relevant physical states during exposure is highly relevant. The latter is a fundamental requirement for aquatic toxicology studies used in a regulatory context and for Life Cycle Impact Assessment. Aligned with the European Commission’s priorities for plastic research, this editorial underscores the importance of integrating life cycle considerations into the assessment of MNPs. The four manuscripts featured in this Research Topic contribute with new perspectives on the mechanisms of MNP toxicity and the role of their physical states during exposure. By advancing our understanding of how these particles interact within ecosystems, the studies support the development of more nuanced regulatory approaches that consider the full life cycle of plastics. Furthermore, by advancing our understanding of MNP behavior in diverse environmental contexts, the studies support the development of regulatory approaches that encompass the full life cycle of plastics. Finally, these contributions emphasize the value of and, arguably, the need for interdisciplinary research in addressing the complexities of plastic pollution, contributing guidance in future evidence-driven regulatory frameworks.
